# Intrapartum Electronic Cigarette Use and Birth Outcomes: Evidence from a Population-Based Study

**DOI:** 10.3390/ijerph21111449

**Published:** 2024-10-31

**Authors:** Michelle Azar, M. Elena Oatey, Michelle H. Moniz, Beth A. Bailey

**Affiliations:** 1 College of Medicine, Central Michigan University, Mt. Pleasant, MI 48859, USA; azar1m@cmich.edu; 2 Women’s Health and Wellness Center, Corewell Health, Grand Rapids, MI 49546, USA; mary.oatey@corewellhealth.org; 3 Department of Obstetrics and Gynecology, University of Michigan, Ann Arbor, MI 48109, USA; mmoniz@med.umich.edu

**Keywords:** vape, combustible cigarette, marijuana, neonatal, adverse effects, birthweight, APGAR, arterial cord pH, assisted ventilation, NICU, neonatal antibiotics

## Abstract

The harms of combustible cigarette (CC) use in pregnancy for fetal development are well studied. Less understood are the potential impacts of newer non-combustible cigarette alternatives, including electronic cigarettes (ECs). Our goal was to examine whether EC use during pregnancy predicts increased risk of adverse birth outcomes. This retrospective cohort study used data from the Obstetrics Initiative (OBI), a statewide collaborative of 70 maternity hospitals. OBI’s clinical registry of data on nulliparous, term, singleton, and vertex fetal presentation pregnancies were from medical records. Three groups of pregnancy cigarette users (Controls (n = 26,394), CC (n = 2216), and EC (n = 493)) were compared on birth outcomes, controlling for background differences. Controls were defined as nonsmokers of ECs or CCs. Compared to the controls, the EC group had significantly lower birth weight, while the CC group had reduced birthweight and greater rates of arterial cord pH < 7.1. Compared to EC users, CC users had higher rates of neonates requiring antibiotics and NICU admission. Growing evidence suggests ECs are not safer alternatives to CCs and use during pregnancy should be discouraged. Additional research is needed, as non-significant trends for increased risk of several adverse neonatal outcomes following EC use were found, potentially significant in larger studies with average risk for adverse pregnancy outcomes and when frequency and timing of EC exposure are considered.

## 1. Introduction

The use of combustible cigarettes (CCs) in pregnancy is the leading preventable cause of poor pregnancy outcomes [1]. The impacts of CC use in pregnancy are well studied and include low birth weight (LBW), small for gestational age (SGA), preterm birth (PTB), infant lung hypoplasia, increased rates of cesarean delivery, birth defects, admission to the neonatal intensive care unit, and fetal and infant deaths [2,3,4]. Less understood are the effects of electronic cigarette (EC) use. The frequency of EC use is increasing, particularly among younger demographics, with an estimated prevalence of 9.2% compared to the prevalence of CC use, which is 3.6% in adults aged 18–24. In the context of pregnant individuals, ECs are used in up to 15% of pregnancies in the US [5]. While the ACOG firmly recommends discontinuation of all forms of smoking, including ECs, during pregnancy [6], studies suggest that the general public believes ECs to be safer in pregnancy than CCs [7]. There are some notable differences between CCs and ECs. CCs contain tobacco and various additives that produce tar and harmful byproducts when combusted, and ECs use a liquid primarily comprised of nicotine, diacetyl, heavy metals, and carbonyl compounds, without any tobacco.

Existing research on the use of ECs in pregnancy involves mostly zebrafish and mouse animal models, and relevance to human offspring in zebrafish models particularly is unclear due to the lack of buffering that would otherwise take place at the fetal–placental interface in mammalian models. However, these studies have identified adverse fertility and offspring development, including deficits in bone growth following exposure to both nicotine-containing and nicotine-free EC aerosols [8]. Cinnamaldehyde flavoring, a common EC offering, was especially harmful to growth, health, and behavioral development [8]. In mammal models, studies have linked exposure to nicotine-containing EC vapor to reduced birthweight [9,10] and adult weight [11,12], while others reported pulmonary [10] and neurological effects, including impacts on motor coordination, learning, behavior, and memory into adulthood [13].

Existing human studies on birth outcomes relative to prenatal EC exposure have largely been performed using the Pregnancy Risk Assessment Monitoring System (PRAMS) database. For example, third-trimester EC use was associated with increased risk for small for gestational age (SGA) [14,15]. Third-trimester exposure was also associated with low birth weight (LBW) across multiple studies utilizing PRAMS [15,16,17,18]. While PRAMS is a national survey soliciting responses from a representative subset of new mothers, non-response and recall bias, as well as social-desirability responding and question formats (for example, questions about ECs are limited to third-trimester use) can limit the validity and generalizability of conclusions of studies using PRAMS. To date, most studies not utilizing PRAMS have reported no differences in birthweight between neonates born to EC-only users and control groups [19,20] but have identified increased risk of preterm birth (PTB) in the EC groups [15,16,17]. Interestingly, one study found that the odds of a pregnancy impacted by the use of only ECs resulting in SGA or PTB were not significantly lower than the group of individuals who used CCs during pregnancy, supporting that ECs are not safer alternatives to CCs [15]. These studies, however, are limited by smaller and more homogeneous samples. Consequently, while existing research suggests that use of ECs in pregnancy may be unsafe, exact findings in relation to PTB, LBW, and SGA are inconsistent across studies. Thus, while the harms of CC use in pregnancy are well studied, data from high-quality studies are lacking regarding comparative use of CCs and ECs on commonly studied adverse neonatal outcomes such as birth size and gestational length, as well as less frequently examined outcomes including low APGAR scores, NICU admissions, supplemental oxygen therapy at birth, infection, and metabolic acidosis.

The goal of the current study was to examine whether EC use during pregnancy increases the risk of a wide variety of birth outcomes compared to non-use and to also examine the differences in outcomes between pregnancy EC and CC use.

## 2. Materials and Methods

### 2.1. Dataset

Data for this retrospective cohort study were obtained from the Obstetrics Initiative (OBI), a quality improvement project funded by Blue Cross Blue Shield of Michigan and Blue Care Network, currently focused on safely lowering the number of cesarean deliveries across the state of Michigan. OBI’s registry includes chart-abstracted data from approximately 70 maternity hospitals in Michigan and includes nulliparous, singleton, term delivery, and vertex (NSTV) births. OBI provided a de-identified dataset of NTSV births between January 2021 and July 2022 in the registry, and our local IRB determined the study was exempt from IRB oversight.

### 2.2. Exposure Definitions

Within the OBI database, EC use and CC use were indicated for participants. OBI allowed the following values in their definition of EC use at any point during pregnancy: e-cigarettes, e-hookah, steam stones, pens, vape pens, and other vaping products. CC use included the following: cigarettes, hookah, chewing tobacco, or any other tobacco-based product during pregnancy. Marijuana use was categorized as positive if there was any marijuana use during pregnancy, regardless of how it was consumed.

For our sample, we excluded those for whom information was unavailable on their EC and CC use status. EC and CC users were defined as individuals who used those products for some or all of their pregnancy, and those who used both (n = 134) were excluded from the current analysis. Participants in the control group were individuals who were definitively identified as non-CC or non-EC users. Patients with any documentation of alcohol or opioid use during pregnancy were excluded from this study.

### 2.3. Outcome Definitions

All available markers of neonatal wellbeing at birth were examined as study outcomes. Birthweight (grams) was included as a continuous outcome. LBW, arterial cord pH, 1- and 5-min APGAR scores, neonatal assisted ventilation at birth and >6 h postpartum, neonatal antibiotics at birth, and NICU admission were all reported as categorical outcomes. LBW was defined as birthweight < 2500 g [21]. Low arterial cord pH was defined as pH < 7.1, indicative of neonatal metabolic acidosis. Low 1 min APGAR scores were defined as scores < 7 and low 5 min APGAR scores as <8. The remaining outcomes were keyed as “yes” vs. “no” depending on if the outcome occurred. Gestational age at delivery was not examined as an outcome due to the dataset including only term births.

### 2.4. Additional Variables

Study population descriptors and model covariates for these first-time mothers included maternal age at delivery, race/ethnicity, pre-pregnancy and delivery weight and BMI, gravidity, parity, diabetes (gestational and preexisting), and hypertension (pregnancy-induced and chronic). Since a consistent indicator of socioeconomic status and related constructs was not available from the medical records at the individual level, we used the Geospatial Research, Analysis & Services Program (GRASP) to provide location information for each study participant in order to calculate the Centers for Disease Control and Prevention Social Vulnerability Index (SVI) for each [22]. SVI indicates the relative vulnerability of the U.S. Census tract in which an individual lives, ranking the tracts on 16 factors including income, education, employment, household composition, disability, minority and language status, and housing and transportation factors. Values represent percentile rankings for each tract that range from 0 to 1, with higher values indicating greater vulnerability.

### 2.5. Data Analyses

Data analyses were completed using IBM SPSS Statistics version 28.0.1.0. Bivariate comparisons between exposure and control groups utilized ANOVA and chi-square analysis. Background variables significantly associated with group status were identified and included as control variables in subsequent regression analyses. Logistic and linear regressions were performed to examine group differences on outcomes controlling for all significant background factors, with adjusted mean differences and adjusted odds ratios reported. A *p* < 0.05 was considered statistically significant for all analyses.

## 3. Results

### 3.1. Background Differences Between Study Groups

The analytic sample contained 26,394 controls, 493 EC-only users, and 2126 CC-only users (Table 1), with the groups differing significantly on all background variables available. All smokers were significantly more likely to be White, non-Hispanic, gain more weight, have a higher mean social vulnerability index, and smoke marijuana during pregnancy compared to the controls. Additionally, those who smoked CCs compared to other participants had a higher mean BMI pre-pregnancy and were significantly more likely to have diabetes. Those who used ECs were significantly less likely to have hypertension than the remaining participants.

### 3.2. Neonatal Outcome Differences Between Study Groups

**Figure 1:**
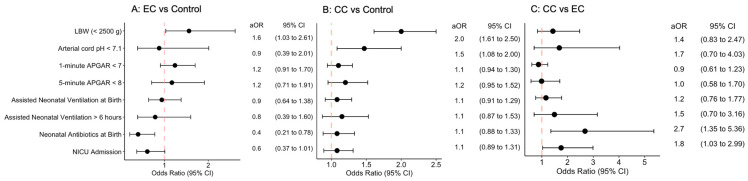
Risk of adverse pregnancy outcomes based on type of cigarette use.

Birthweight was analyzed as both a continuous and categorical variable. All other variables were analyzed as categorical variables, and adjusted odds ratios, controlling for significant background variables, and corresponding confidence intervals can be seen in Figure 1 for the between-group comparisons. Compared to the control group, EC users gave birth to babies on average 109 g (95% CI −151 to −68) lighter than those who did not use any cigarettes during pregnancy. Participants in this group were also 60% more likely than the controls to have a newborn classified as LBW. The risk for NICU admission was significantly decreased for those born to EC users. When evaluating risks of low 1 and 5 min APGAR scores, neonatal metabolic acidosis, and necessity of neonatal antibiotics and assisted ventilation both at birth and >6 h postpartum, there were no statistically significant differences between the EC and control groups.

In comparing the CC group to the controls, those who used CCs had a doubled risk of an LBW baby. The CC group was almost 50% more likely than the controls to have a newborn with metabolic acidosis, which was statistically significant. Similarly, the risk of low 1 and 5 min APGAR scores, needing neonatal antibiotics, NICU admission, and needing neonatal assisted ventilation at birth and for >6 h postpartum were increased for babies born to CC users, though not statistically significant.

When observing the exposure groups against one another, there was no significant difference in birthweight (adjusted mean −39 g, 95% CI −88 to 7), risk of giving birth to an LBW baby, or risk of neonatal metabolic acidosis. Babies born to CC users were more than 2.5 times more likely to require neonatal antibiotics, however, and over 1.5 times more likely to be admitted to the NICU than babies born to EC users. All effects noted here were statistically significant.

Panels A–C indicate risk of adverse pregnancy outcomes for the above group comparisons, where an adjusted odds ratio (aOR) >1 indicates an increased risk for the EC group compared to the control group, CC group compared to the control group, and CC group compared to the EC group, respectively. The results are from linear and logistic regression analyses controlled for all factors in Table 1.

## 4. Discussion

The findings from this large population-based study of full-term infants demonstrate that use of ECs during pregnancy, compared to no use of ECs or CCs, was associated with significantly decreased birthweight and a trend toward increased risk of lower 1- and 5-min APGAR scores. The use of CCs in pregnancy was also associated with reduced birthweight, along with greater rates of arterial cord pH less than 7.1. When comparing the CC and EC groups to one another, CC use in pregnancy was found to have a significantly higher risk of neonates requiring antibiotics at birth and NICU admission, but no other significant differences were observed.

To our knowledge, this is the first large population-based analysis assessing adverse neonatal outcomes associated with use of ECs in pregnancy that looked beyond gestational age and size at birth to examine other neonatal outcomes including low APGAR score, metabolic acidosis, respiratory distress, infection, and NICU admission. Like many other studies, ours demonstrates decreased birthweight as a significant risk associated with both electronic and combustible cigarette use. While the effect was larger when quantifying the average decrease in birthweight among babies born to CC users, the decrease in birthweight was still significant for babies born to EC users when compared to the controls, indicating use of ECs in pregnancy may be independently harmful and not an appropriate alternative to CCs. The developmental sequelae for most LBW babies are poor, with increased incidence of cognitive and attentive deficits, as well as problems with neuromotor functioning into adolescence [23], indicating long-term detrimental effects that could follow EC exposure during gestation.

Our study found a trend toward increased risk for low 1- and 5-min APGAR scores in both the EC and CC groups, with the risk being most elevated in the CC group. APGAR scores are generally regarded as markers for how well babies tolerated birth and how well they are adjusting to being outside of the womb. While APGAR scores have not been clinically useful in predicting individual neonatal mortality or longer-term outcomes, a 5 min score of 3 or less confers increased risk of neonatal mortality at the population level as well as an increased relative risk of cerebral palsy [24]. While our cutoff for defining a low APGAR score was higher, studies show that scores below 7 are concerning for neonatal hypoxic-ischemic encephalopathy, meconium aspiration, respiratory distress, and mortality [25,26,27,28], indicating that scores below this level may have implications for longer-term detrimental effects.

Interestingly, increased risk for neonatal assisted ventilation, antibiotic administration, and NICU admission in the CC group when compared to the controls was nonsignificant in this study, in contrast to some other studies where these effects have been found. Previous literature on the subject has described associations between maternal CC use in pregnancy and increased risk of low birth weight, neonatal NICU admission, infection requiring antibiotics, and respiratory distress requiring assisted ventilation [29,30,31,32,33,34]. Our population was inherently low-risk since participants who delivered preterm, were non-NSTV, and who experienced fetal death were not included in OBI’s sampling. Thus, individuals who had adverse outcomes associated with both EC and CC use may have been eliminated from the original dataset, suggesting that our findings may conservatively estimate the true effect among higher-risk populations, including those with combined EC and CC use (who were excluded from the analysis) or those with prematurity (who were not included in our dataset).

Our study has many strengths. To our knowledge, it is the first large, population-based study examining birth outcomes following EC use in pregnancy using data objectively captured in electronic medical records, rather than data from those who choose to be in the study and self-report all variables, such as is the case with PRAMS. In addition, PRAMS only captures third-trimester EC use, while our dataset included EC use in any part of pregnancy. Use of ECs in the first trimester alone has been associated with adverse neonatal outcomes including PTB, LBW, SGA, neonatal assisted ventilation, NICU admission, and infant death [35]. Including individuals who used ECs at any point in pregnancy, including those that quit during pregnancy, captures a more representative population of EC use in pregnancy rather than limiting it to third-trimester use and may also lead to the discovery of more associated adverse outcomes. Further, PRAMS does not include many outcomes analyzed in this study, including arterial cord pH, APGAR scores, neonatal assisted ventilation, neonatal antibiotic administration, and NICU admission, many of which predict long-term health and developmental issues. Additionally, most published research on this topic not using PRAMS datasets is poorly generalizable due to smaller and homogenous samples.

A further strength of our study is that we included participants who used marijuana in pregnancy concomitantly with cigarettes. With expanding legalization, marijuana usage rates have been increasing and research suggests that those who use ECs are significantly more likely to use marijuana concomitantly or later [36,37]. Thus, we designed our study to include these individuals as more representative of the general population of EC users, adding validity to this study and increasing power by including more participants. Further, a recent study found that concomitant use of tobacco and marijuana during pregnancy was not associated with additional risks of poor neonatal outcomes compared to tobacco use alone [38], supporting that significant findings from our study are not explained by in utero marijuana exposure alone and as additionally indicated by significant effects after control for marijuana use. Our study is additionally strengthened by excluding participants who used other substances beyond marijuana, including alcohol and opioids, which have well-established effects on newborn outcomes and could decrease the validity of our findings. Thus, our study utilizes a larger dataset with expanded inclusion of birth outcomes and pregnancy EC use before the third trimester and represents concomitant use of marijuana while controlling for effects of other substances, all of which strengthen the current study and make findings more generalizable than the results of previous studies.

This study should be interpreted considering some limitations. First, there were fewer EC-only users in comparison to CC-only users and the control groups. As ECs are still new to the market, we expect future studies will have proportionately more EC users as sales of ECs continue to grow. Further, our data were not granular enough to examine type of ECs used, frequency of use, and duration and timing of use in pregnancy, which are likely relevant to the findings. Animal studies indicated potential harm to cardiovascular development from flavorings and other toxicants in nicotine-free ECs [39]. Thus, there are independent harms associated with the use of nicotine-free ECs, which would be valuable to ascertain in a human-based study. In future studies, it would be beneficial to observe outcomes relative to type, frequency, and duration of use. Lastly, as our dataset only contained live, full-term, NSTV births, the low-risk nature of the participants may have impacted findings including not being able to assess associations with preterm delivery or stillbirth. Thus, cases for whom EC use led to adverse outcomes were potentially eliminated. This low-risk sample may also be why, unlike other studies, we identified fewer adverse outcomes following tobacco use in pregnancy. This suggests that EC effects may be larger in more generalized samples as well, but our study does show that EC and CC use in pregnancy predict adverse outcomes even when gestational age at delivery is not impacted.

## 5. Conclusions

While use of ECs in pregnancy reduced birth weight in this study, most adverse newborn outcomes examined were not significantly predicted by EC use. Additional study is needed, as non-significant trends toward poorer outcomes for EC users were evident and potentially significant with larger samples that include higher-risk deliveries or when information on the amount and timing of EC exposure is considered. These findings suggest that detailed prospective studies, especially those that examine if there are subsets of women for whom EC use in pregnancy is especially risky, are needed, and further reinforce that the lack of significant findings related to prenatal substance exposure should be carefully scrutinized before conclusions about lack of harm are made. Finally, our findings can motivate and inform efforts to educate and counsel about EC use during prenatal care, including making sure that perinatal quality improvement initiatives focused on smoking cessation also address EC use.

## Figures and Tables

**Table 1:** Participant background factors by cigarette use status.

	Controls (n = 26,394)	Electronic Cigarette Only (n = 493)	Combustible Cigarette Only (n = 2126)	*p*
Race (% White, non-Hispanic)	68.5%	84.9%	79.5%	<0.001
Social Vulnerability Index ^a^	0.436	0.453	0.489	<0.001
Pre-pregnancy BMI (kg/m^2^)	27.5	27.4	28.6	<0.001
Pregnancy weight gain (lbs.)	31.7	36.3	34.6	<0.001
Diabetes (pre-existing or gestational, %)	8.8%	8.7%	10.3%	0.045
Hypertension (chronic or pregnancy-induced, %)	19.8%	16.2%	21.9%	0.009
Pregnancy marijuana use (%)	8.9%	46.9%	48.4%	<0.001

Note: Analyses were one-way ANOVA for continuous factors and chi-square analysis for categorical factors, with reported *p* values from 2-tailed analysis. ^a^ CDC Social Vulnerability Index value for the census tract of the participant’s residence. Values can range from 0 to 1, with higher scores indicating greater social vulnerability.

## Data Availability

Restrictions apply to the availability of these data. Data were obtained with written approval from OBI with the agreement that the data would not be distributed without consent. Any requests for data acquisition must be placed with OBI and are only available directly from that organization.
